# Severe and Enduring' Stage in Anorexia Nervosa: Comparing Eating Attitudes, Impairment and Associated Psychopathology

**DOI:** 10.3389/fnut.2022.867401

**Published:** 2022-03-28

**Authors:** Rita Ramos, Ana Vaz, Tânia F. Rodrigues, Ana Pinto-Bastos, Isabel Brandão, António Neves, Eva Conceição, Paulo P. P. Machado

**Affiliations:** ^1^Psychotherapy and Psychopathology Research Lab - CIPsi, School of Psychology, University of Minho, Braga, Portugal; ^2^Psychiatry Department, Hospital of S. João, Faculty of Medicine, University of Porto, Porto, Portugal; ^3^Eating Disorders Unit, Psychiatry Department, Hospital of Santa Maria, Faculty of Medicine, University of Lisbon, Lisbon, Portugal

**Keywords:** Anorexia Nervosa, duration of illness, clinical impairment, severe-enduring criteria, staging

## Abstract

This study aimed to assess differences in eating attitudes, impairment, and related psychopathology at treatment presentation for patients with “Non-severe and enduring Anorexia Nervosa” (illness duration of <7 years) and patients with “severe and enduring Anorexia Nervosa” (illness duration of 7 years or more). One hundred and thirty-nine patients diagnosed with Anorexia Nervosa participated in this study. Participants were interviewed with the Eating Disorder Examination (EDE) and asked to complete several questionnaires at the end of the first treatment appointment. We also explored differences at treatment presentation by considering alternative criteria to define groups, namely a composite of illness duration and clinical impairment (≥16 CIA total score). No differences were found when comparing participants based on illness duration. However, when participants were classified into a different classification scheme: “Non-severe and enduring Anorexia Nervosa” (illness duration <7 years and a CIA total score <16) vs. “severe and enduring Anorexia Nervosa” (illness duration ≥7 years and CIA total score ≥16), significant differences were found in terms of eating pathology, depressive symptomatology, psychological distress, and emotion dysregulation. Further research is needed to better understand the role of illness duration and clinical impairment in informing the course of AN.

## Introduction

Anorexia Nervosa (AN) is a severe psychiatric illness associated with various medical complications that arise as a result of weight loss and malnutrition, as well as with increased mortality rates (1, 2). Even receiving treatment, many patients do not recover or remain symptomatic. Accordingly, a recent study (3) found recovery rates of 62.8% at 22-year follow-up with ~40% remaining symptomatic.

In recent years, a staging model of Eating Disorders (ED) has been formulated to better understand the different stages of illness over time. This model categorizes ED psychopathology into a set of severity stages (i.e., high risk, prodromal, full syndrome, and severe and enduring) associated with the development of a form of neuroprogression that results from malnutrition and abnormal eating patterns (4). In AN, the severe and enduring stage (SE-AN) has been receiving increasing attention in the literature; however, an accepted definition of this stage is still lacking (5). Common criteria used to define this final stage of the staging model include previously failed treatment attempts, underweight body mass (6), and an illness duration of at least 7 years (7), but a consensual, empirically-based definition of this stage is still an important research goal.

While some studies found significant differences in several clinical outcomes between patients with SE-AN and non-SE-AN (8), other studies found no difference when the only criterion for the distinction of the two categories is the temporal criterion (9, 10). These findings have contributed to controversy over the clinical utility of a SE-AN classification based solely on the criterion of illness duration.

Functional impairment has also been positively associated with severity in patients with EDs (11). Glasofer et al. (12) found that severity at discharge from an ED inpatient treatment was positively associated with clinical impairment at 5-year follow-up in patients with AN. Similarly, a recent study using network analysis (13) indicated that the centrality of symptoms in the network was associated with clinical impairment after treatment. Thus, clinical impairment should be investigated as a marker of the severity of Anorexia Nervosa in longer durations, since it may differentiate individuals with an important compromise secondary to ED psychopathology regarding personal, cognitive, and social dimensions that are important as therapeutic targets in severe and enduring states. The development of a consensual definition of the severe and enduring form of AN is critical, as it would facilitate the development of evidence-based treatment approaches for this population, guide the intervention, and determine which patients would benefit from treatments tailored for this population (5).

The present study aims to deepen the knowledge around the conceptualization of AN psychopathology across different severity stages, and to explore the utility of illness duration as a criterion to distinguish more severe and enduring courses. Specifically, we intend to assess differences in demographic and clinical characteristics, eating attitudes, clinical impairment, and related psychopathology at treatment presentation for patients categorized as being in the “non-SE-AN” stage vs. those in the SE-AN stage. For this purpose, a definition of <7 years and 7 years or more was adopted, respectively, since the cut-off of 7 years has been the most frequently used in previous research (6). We hypothesize that patients with longer illness duration will present greater ED psychopathology, clinical impairment, depressive symptomatology, emotion dysregulation, and psychological distress than the group with an illness duration of <7 years. We also intend to explore differences at treatment presentation considering an alternative classification scheme to define groups, namely a composite of illness duration and clinical impairment, since its established association with severity in ED patients (14).

## Materials and Methods

### Participants

Participants were 139 patients (128 female, and 11 male) diagnosed with Anorexia Nervosa recruited from two hospital units specialized in treating eating disorders in the North and Center of Portugal, either inpatient or outpatient. All participants were referred to the study by their psychiatrist at the end of the first appointment of the current treatment and assessed that same day. Participants' age ranged from 13 to 53 years (*M* = 23.83, *SD* = 8.70). Mean BMI (kg/m^2^) was 16.07 (*SD* = 2.25). One hundred and five participants were diagnosed with AN-Restricting subtype and 34 participants with AN-Binge Eating/Purging subtype. The majority (82.0%, *n* = 114) were single, 9.4% (*n* = 13) were married, 1.4% (*n* = 2) divorced, and 2.2% (*n* = 3) lived with a partner. About half of the sample were students (*n* = 68, 48.9%), 21.6% (*n* = 30) were employed, 7.9% (*n* = 11) unemployed, 2.9% (*n* = 4) had no occupation, 0.7% (*n* = 1) were retired, and 1.4% (*n* = 2) were on sick leave. The mean duration of AN was 62.00 months (*SD* = 74.81).

One hundred and six participants (76.3%) were classified as having an illness duration of <7 years, and 33 (23.7%) participants as having an illness duration of 7 years or more, according to a semi-structured clinical interview to address the first time participants met the criteria for the diagnosis of Anorexia Nervosa. Illness duration ranged between 3 and 84 months (*M* = 28.66, *SD* = 21.80) for the group with an illness duration of <7 years, and between 96 and 456 months (*M* = 169.09, *SD* = 84.07) for the group with an illness duration of 7 years or more.

### Procedure

The present study is part of a larger naturalistic longitudinal study that focuses on treatment monitoring of ED patients in outpatient and inpatient treatment settings.

During the first treatment appointment, all participants were interviewed by a psychiatrist to obtain clinical history information and for diagnostic purposes. Those who met the criteria for the diagnosis of Anorexia Nervosa and agreed to participate in the study were interviewed by clinical psychologists trained in ED assessment and treatment, using the Eating Disorders Examination (15) and a clinical interview for demographic information and clinical ED-related history. Afterward, participants completed a series of self-report questionnaires to assess variables of interest.

Written informed consent was obtained from all participants or their parents if aged <18 years. The present study was approved by the ethical committees of all the institutions involved.

### Definition of Illness Duration

Each participant was classified according to AN illness duration based on a semi-structured clinical interview to assess clinical history, namely weight history, dieting behavior and clinical related variables (15) conducted by clinical psychologists specialized in ED treatment and assessment. To obtain a more detailed description of each patient's clinical history, participants were also interviewed with a complementary semi-structured interview (16), which allowed the collection of additional information about the history of the illness (e.g., the age of onset of the most significant ED behaviors), and that was then adapted for the definition of illness duration. This variable was defined as the time elapsed between the onset of AN (i.e., the first time participants presented symptoms clinically indicative of the presence of AN diagnosis) and the time of the study assessment.

The sample was divided into two groups: the group with “non-SE-AN” (i.e., illness duration of <7 years) and the group with SE-AN (i.e., illness duration of 7 years or more), to mirror the most accepted definitions in the literature.

### Measures

Eating Disorders Examination (EDE) (15): is a semi-structured interview that aims to assess eating disorder related psychopathology and dysfunctional eating behaviors. Only the diagnostic items were used in the present study.

Eating Disorder Examination-Questionnaire (EDE-Q) (17): is a 28-item self-report measure used to assess eating disorder symptoms and associated psychopathology over the last 28 days. It comprises four subscales (restraint, eating concern, shape concern, and weight concern) and a total score. The Portuguese version of the EDE-Q has demonstrated good internal consistency, with Cronbach's alphas ranging from 0.83 to 0.96 (18). Cronbach's α for our sample was 0.93 for the global score.

Outcome Questionnaire-45 (OQ-45) (19): is a 45-item self-report measure designed to assess patients' progress in a therapeutic setting but can also be used as a measure of psychological distress. The OQ-45 includes three subscales: Symptom distress (SD), Interpersonal Relationships (IR), and Social Role Functioning (SR). It was used as a baseline measure of symptoms of psychological distress. The Portuguese version has demonstrated good internal consistency (Cronbach's α = 0.93). Cronbach's α for our sample was 0.76 for the global score.

Beck Depression Inventory (BDI) (20): is a 21-item self-report measure developed to assess cognitive, affective, and somatic symptoms of depression over the last month. The Portuguese version has demonstrated excellent internal consistency (Cronbach's α = 0.92) (21). Cronbach's α for our sample was 0.89.

Clinical Impairment Assessment (CIA) (22): is a 16-item self-report measure that assesses clinical impairment secondary to eating disorders in three domains (personal, social, and cognitive) over the last 28 days. The global score ranges from 0 to 48, and higher scores represent more impairment. The Portuguese version has demonstrated good internal consistency (Cronbach's α = 0.96) (23). Cronbach's α for our sample was 0.95 for the global score.

Difficulties in Emotion Regulation Scale (DERS) (24): is a 36-item self-measure that assesses patients' response to negative emotional states. The global score ranges from 36 to 180, and higher scores represent greater difficulties in emotion regulation. The Portuguese version has demonstrated excellent internal consistency (Cronbach's α = 0.93) (25). Cronbach's α for our sample was 0.90 for the global score.

### Statistical Analysis

All statistical analyses were conducted using the IBM SPSS Statistics software (version 27.0).

For the characterization of the sample, descriptive statistics were performed. Differences between groups with non-SE-AN (i.e., <7 years duration) and SE-AN (i.e., ≥7 years duration) were estimated with Mann-Whitney tests for age, body mass index, weight suppression (i.e., the difference between highest and current weight), and the number of hospitalizations. Chi-square tests were used for categorical data (previous treatments, overweight history, AN severity, AN subtype, previous eating disorder treatment, and treatment setting).

Analysis of Covariance (ANCOVA) was conducted to analyze differences between groups regarding eating attitudes and related psychopathology (EDE-Q, OQ-45, BDI, CIA, and DERS score). Age was introduced as a co-variable in all analyses. The same analysis was performed for groups with clinical impairment <16 and clinical impairment ≥16.

Differences between inpatient and outpatient groups regarding variables under study were tested using Univariate Analysis of Variance (ANOVA), and no differences were found.

We also explored differences at treatment presentation by considering an alternative SE-AN classification scheme, namely a combination of illness duration and clinical impairment (CIA Total score). For this purpose, ANCOVA models were conducted with the group (illness duration <7 and clinical impairment <16; illness duration <7 and clinical impairment ≥16; and illness duration ≥7 and clinical impairment ≥16) as the independent variable (IV), the variables under study as dependent variables (DV), and age as a covariate. The group “illness duration ≥7 and clinical impairment <16” was not included in the analysis, since the small number of participants included in this group (*n* = 3). *Post hoc* Bonferroni tests were conducted for significant ANCOVAs. Exploratory analysis was also performed by considering a combination of illness duration and depressive symptomatology (BDI Total score ≥13), and no statistically significant differences were found.

## Results

Differences in clinical and demographic characteristics between “non-SE-AN” and SE-AN groups are summarized in Table 1.

**Table 1:** Group differences in clinical and demographic characteristics, comparing “Non-SE-AN” and “SE-AN” groups.

		**Non-SE-AN (<7 years duration) (*n* = 106) M (SD)**	**SE-AN (≥7 years duration) (*n* = 33) M (SD)**	**Statistics**	***p***
Age (years)	Range: 13–53	21.09 (6.61)	32.61 (8.88)	Z = −6.769	<0.001^***^
BMI	Range: 11.20–24.14	16.05 (2.24)	16.14 (2.30)	Z = −0.124	0.902
Weight suppression (kg)	Range: 0–45.10	15.08 (9.07)	17.64 (11.04)	Z = −1.014	0.311
Number of hospitalizations	Range: 0–15	0.32 (0.76)	1.73 (3.38)	Z = −2.789	0.005^**^
Previous Treatments	Yes No	75 (96.2%) 3 (3.8%)	20 (90.9%) 2 (9.1%)	χ2 (1) = 0.994	0.319
Overweight history	Yes No	20 (19.6%) 82 (80.4%)	8 (28.6%) 20 (70.1%)	χ2 (1) = 1.044	0.307
Severity	Mild Moderate Severe Extreme	32 (30.2%) 18 (17.0%) 19 (17.9%) 37 (34.9%)	9 (27.3%) 5 (15.2%) 9 (27.3%) 10 (30.3%)	Cramer's V = 0.099 χ2 (3) = 1.373	0.712
Subtype	Restricting Binge Eating/Purging	84 (79.2%) 22 (20.8%)	21 (63.6%) 12 (36.4%)	χ2 (1) = 3.318	0.069
Previous ED Treatment	Psychologist Psychiatrist Both Pedopsychiatry Other None	18 (23.1%) 27 (34.6%) 22 (28.2%) 1 (1.3%) 0 (0.0%) 10 (12.8%)	1 (4.5%) 6 (27.3%) 10 (45.5%) 0 (0.0%) 1 (4.5%) 4 (18.2%)	χ2 (5) = 9.157	0.103
Treatment setting	Inpatient Outpatient	22 (20.8%) 84 (79.2%)	10 (30.3%) 23 (69.7%)	χ2 (1) = 1.295	0.255

*N = 139*.

*M, Mean; SD, Standard deviation; BMI, Body Mass Index; ED, Eating Disorder. Severity was calculated according to the DSM-5 severity criterion based on body mass index cut-points*.

** *p <0.01*.

*** *p <0.001*.

Findings revealed a significant difference in the number of hospitalizations between the groups, with higher values for the SE-AN group. There was also a significant difference in age between groups, as expected.

Differences in eating attitudes and related psychopathology across “non-SE-AN” and “SE-AN” groups are summarized in Table 2. The percentage of participants with a CIA total score ≥16 was 75.2% in the group with an illness duration of <7 years, and 90% in the group with an illness duration of 7 years or more. No statistically significant differences were found between groups.

**Table 2:** Differences between “Non-SE-AN” and SE-AN groups regarding eating attitudes and related psychopathology.

	**Non-SE-AN (<7 years duration) (*n* = 106) M (SD)**	**SE-AN (≥7 years duration) (*n* = 33) M (SD)**	***Z***	***p***	***Partial η2***
EDE-Q Total Score	2.87 (1.65)	2.73 (1.69)	0.605	0.438	0.005
OQ-45 Total Score	75.18 (32.59)	82.05 (26.79)	0.558	0.457	0.005
BDI	21.50 (11.62)	20.89 (11.17)	0.000	0.995	0.000
CIA Total Score	23.74 (13.96)	27.20 (12.02)	0.939	0.335	0.009
DERS Total Score	98.01 (33.42)	93.96 (29.70)	0.183	0.670	0.002

*N = 139*.

*EDE-Q, Eating Disorder Examination-Questionnaire; OQ-45, Outcome Questionnaire-45; BDI, Beck Depression Inventory; CIA, Clinical Impairment Assessment; DERS, Difficulties in Emotion Regulation Scale*.

*Exploratory analysis of the differences at treatment presentation when a composite of illness duration and clinical impairment was considered:*

Several exploratory analyses of the differences at treatment presentation in eating attitudes and related psychopathology when a composite variable of illness duration and clinical impairment was considered were tested.

ANCOVAs revealed significant differences between the groups in terms of eating pathology, depressive symptomatology, psychological distress, and emotion dysregulation (Table 3). *Post hoc* tests showed that the group “illness duration ≥7 years and clinical impairment ≥16” presented significantly higher eating psychopathology (*p* < 0.001), depressive symptomatology (*p* < 0.001), psychological distress (*p* < 0.001), and emotion dysregulation (*p* < 0.01) than the “illness duration <7 and clinical impairment <16” group. The group “illness duration <7 and clinical impairment ≥16” presented significantly higher eating psychopathology (*p* < 0.001), depressive symptomatology (*p* < 0.001), psychological distress (*p* < 0.001), and emotion dysregulation (*p* < 0.001) than the “illness duration <7 years and clinical impairment <16” group. No significant differences were found between the “illness duration <7 and clinical impairment ≥16” and the “illness duration ≥7 years and clinical impairment ≥16” groups.

**Table 3:** Differences between groups in terms of eating attitudes and related psychopathology when a composite of illness duration and clinical impairment was considered.

	***Univariate results***						***Post-hoc***		***Effect size***
	**Group A (Illness duration <7 and clinical impairment <16) *n* = 26 M (SD)**	**Group B (Illness duration <7 and clinical impairment ≥16) *n* = 79 M (SD)**	**Group C (Illness duration ≥7 and clinical impairment ≥16) *n* = 27 M (SD)**	***Z***	***p***	***Partial η2***	***≠ between groups***	***p* value**	***Cohen's d***
EDE-Q	1.12 (0.83)	3.53 (1.40)	3.24 (1.53)	33.564	<0.001^***^	0.371	Group A < Group B Group A < Group C	<0.001^***^ <0.001^***^	1.90 1.78
OQ-45	41.08 (19.51)	88.94 (26.16)	86.89 (26.31)	34.857	<0.001^***^	0.404	Group A < Group B Group A < Group C	<0.001^***^ <0.001^***^	1.96 2.04
BDI	10.46 (6.60)	25.54 (10.41)	22.45 (10.49)	22.541	<0.001^***^	0.258	Group A < Group B Group A < Group C	<0.001^***^ <0.001^***^	1.58 1.40
DERS	68.00 (24.25)	109.87 (29.17)	101.50 (28.07)	19.650	<0.001^***^	0.278	Group A < Group B Group A < Group C	<0.001^***^ 0.003^**^	1.50 1.29

*N = 132*.

*EDE-Q, Eating Disorder Examination-Questionnaire; OQ-45, Outcome Questionnaire-45; BDI, Beck Depression Inventory; DERS, Difficulties in Emotion Regulation Scale*.

** *p <0.01*.

*** *p <0.001*.

## Discussion

The present study aimed to explore the differences in treatment presentation between patients with non-SE-AN (defined as an illness duration of <7 years) and patients with SE-AN (defined as an illness duration of 7 years or more) regarding clinical characteristics, namely eating attitudes, clinical impairment, and related psychopathology.

Differences between groups were found in terms of the number of previous hospitalizations, which were higher for the SE-AN group. Accordingly, the number of hospitalizations has been previously considered an indicator of the enduring aspect of the SE-AN stage (26). In addition, a recent study from Hay and Touyz (27) proposed that unsuccessful treatments, namely the exposure to at least two evidence-based treatments, constitute a criterion for SE-AN definition. No differences were found between groups regarding eating attitudes and related psychopathology at clinical presentation. Time *per se* did not seem to differentiate patients with Anorexia Nervosa at treatment presentation. These results are in line with a recent study from Calugi et al. (9) which reported that patients with SE-AN (defined as the duration of illness of at least 7 years) and non-SE-AN displayed similar improvements in BMI, eating disorder symptoms, and general psychopathology following enhanced cognitive behavioral therapy (CBT-E), at Post-treatment and 12-month follow-up. Wildes et al. (10), using structural equation modeling, also reported that illness duration did not differentiate groups with higher and lower severity profiles in their sample, but eating disorders behaviors and quality of life did.

However, when we considered in the exploratory analysis both illness duration and clinical impairment as markers of severity and “enduringness”, there were significant differences between groups in terms of eating psychopathology, depressive symptomatology, symptom distress, and emotion dysregulation, but only regarding the group with less illness duration and severity (i.e., illness duration of <7 years and a clinical impairment below the clinical cut-off). These findings did not allow to differentiate groups with similar functional impairment regardless of illness duration, reinforcing that illness duration *per se* may not provide sufficient information for differentiating severity profiles. However, in our sample 90% of those with a duration of illness >7 years were above the clinical impairment cut-off, suggesting that long duration of illness can be considered a proxy of severity. On the other hand, shorter duration of illness should not be associated with presumed lowest severity. Groups did not significantly differ regarding BMI. Accordingly, only the group with a shorter illness duration and less impaired differed significantly in eating and weight attitudes, psychological distress, depressive symptoms and emotion regulation difficulties at treatment presentation.

Interestingly, our study's results point out that both groups “illness duration ≥7 and CIA total score ≥16” and “illness duration <7 and CIA total score ≥16” showed poorer scores on all measures as compared to the “illness duration <7 and CIA total score <16” group, suggesting that clinical impairment may have an important role as a marker of severity in AN. This is in line with previous studies on the association between clinical impairment and severity in patients with ED (11).

It is important to note that differences between groups with CIA total score <16 and CIA total score ≥16 were also explored, and statistically significant differences were found for all measures in study. Nevertheless, in the present study the proportion of patients with impairment above the clinical cut-off was higher for those with higher duration of illness, emphasizing the possible interrelation between severity and longer illness duration. This overlap is important to consider in future research, as well as the potential influence that a longer course of illness may itself have on severity. Future studies should consider longitudinal assessment and treatment outcomes in these subgroups of patients with AN.

This study's results do not support the study from Ambwani and colleagues (8) that found statistically significant differences at baseline regarding eating disorder symptomatology when considering a SE-AN classification based on both illness duration and emotional distress as a marker of severity, but different comparison groups were used (illness duration <3 years vs. illness duration ≥7 and Depression, Anxiety and Stress Scale (DASS) total ≥60). Our study uses different classification groups adding a classification based on less duration but equal impairment, which did not present differences regarding eating attitudes and associated psychopathology.

In conclusion, this study does not provide support for a SE-AN classification based solely on an illness duration of 7 years or more, and highlights the need to further explore the validity of a SE-AN conceptualization beyond the indicators of the “enduring” component (26). Accordingly, Hay and Touyz (27) recently proposed the exposure to at least two evidence-based treatments, a duration of AN >3 years, and a persistent state of restriction, low weight and over-evaluation of weight and shape with functional impairment as testable criteria for SE-AN. Also, a recent study found statistically significant differences regarding ED symptomatology when considering a SE-AN classification based on both illness duration and emotional distress as a marker of severity (8), which should also be further explored and considered in future research around the SE-AN classification.

Future studies should explore the characterization of the severe stage in long duration Anorexia Nervosa which can differentiate patients and individualized care. In fact, therapeutic outcomes in patients with severe and enduring courses are divergent and an important research goal. For example, a recent study (28) found that illness severity and duration did not predict treatment outcome in CBT-E. Similarly, another study that compared treatment outcomes in patients with illness duration shorter or >7 years indicated similar improvements after discharge and at 12 month (9). In opposition, in a study from Wild et al. (29), the authors found that illness duration was a negative predictor of treatment outcome.

Future research is needed to better understand the link between illness severity, clinical impairment, and illness duration in AN, namely in longitudinal studies with larger sample sizes. This study is cross-sectional and longitudinal data are needed to inform the predictive value of the duration of illness on illness severity and enduring stage. Also, in the present study, we did not include variables such as the presence of psychiatric comorbidity (e.g., personality disorders, mood, and anxiety disorders) or neurocognitive domains, like uncertainty intolerance traits, negative thought processes (e.g., rumination), and habit formation, which could differentiate groups and should be included in future studies. Longer illness duration stages have also been associated with neuroprogressive changes (4), which should be considered in future research. Several limitations should be considered when interpreting the present findings, namely the heterogeneity of the sample in terms of the gender and AN subtype, as they can make the results less generalizable. Moreover, the groups “non-SE-AN” and SE-AN, as well as the groups “illness duration <7 and clinical impairment <16”, “illness duration <7 and clinical impairment ≥16” and “illness duration ≥7 and clinical impairment ≥16” are not balanced in terms of sample size, which should be considered when interpreting this study's results. Future studies should replicate these analyses with a more homogeneous number of participants in each group and with a larger sample size.

Despite these limitations, it is important to note that our sample consisted of patients with AN who were starting specialized ED treatment, which can be considered a strength of the study.

In conclusion, the present study is an important first step to explore the clinical utility of illness duration at presentation for treatment in AN, as well as to consider clinical impairment as a marker of severity. Our results highlight that patients with longer duration of illness and higher impairment secondary to ED may require more individualized and specific treatment. In addition, it highlights the need to ensure a close monitoring and fast access to treatment to these subgroups of patients since an early stage in order to prevent the development of an enduring course with associated functional impairment. Additionally, addressing impairment secondary to the ED can be used as an important clinical strategy to motivate patients to change. Further research is needed to understand illness's progress through different severity stages and the factors associated with the course of illness.

## Data Availability Statement

The raw data supporting the conclusions of this article will be made available by the authors, without undue reservation.

## Ethics Statement

The studies involving human participants were reviewed and approved by Ethics Committee for Research in Social and Human Sciences (CEICSH). Written informed consent to participate in this study was provided by the participants' legal guardian/next of kin.

## Author Contributions

RR, AV, and PM took primary responsibility for the manuscript, including reviewing relevant literature, and drafting the paper for publication. AP-B and TR were responsible for data collection, participant recruiting, contributed to its analysis, and interpretation. EC contributed to its analysis and interpretation. IB and AN conducted the diagnostic interviews. All authors assisted with the literature review, editing of the manuscript, contributed to the planning, design of the study, read, and approved the final manuscript. All authors contributed to the article and approved the submitted version.

## Funding

This work was conducted at the Psychology Research Center (PSI/01662), School of Psychology, University of Minho, and partially supported by the Portuguese Foundation for Science and Technology and the Portuguese Ministry of Science, Technology and Higher Education (UID/PSI/01662/2019), through the national funds (PIDDAC), and a grant by the Portuguese Foundation for Science and Technology and the Portuguese Ministry of Science, Technology and Higher Education through national funds and co-financed by FEDER through COMPETE2020 under the PT2020 Partnership Agreement (POCI-01-0145-FEDER-028145) to PM. EC was financed through grants 2020.01538.CEECIND and PTDC/PSI-GER/28209/2017. The funding body had no role in the design, collection, analysis, and interpretation of data; the writing of the manuscript; or the decision to submit the manuscript for publication.

## Conflict of Interest

The authors declare that the research was conducted in the absence of any commercial or financial relationships that could be construed as a potential conflict of interest.

## Publisher's Note

All claims expressed in this article are solely those of the authors and do not necessarily represent those of their affiliated organizations, or those of the publisher, the editors and the reviewers. Any product that may be evaluated in this article, or claim that may be made by its manufacturer, is not guaranteed or endorsed by the publisher.
