# Corrigendum: Severe and enduring' stage in anorexia nervosa: comparing eating attitudes, impairment and associated psychopathology

**DOI:** 10.3389/fnut.2023.1292404

**Published:** 2023-09-22

**Authors:** Rita Ramos, Ana Vaz, Tânia F. Rodrigues, Ana Pinto-Bastos, Isabel Brandão, António Neves, Eva Conceição, Paulo P. P. Machado

**Affiliations:** ^1^Psychotherapy and Psychopathology Research Lab - CIPsi, School of Psychology, University of Minho, Braga, Portugal; ^2^Psychiatry Department, Hospital of S. João, Faculty of Medicine, University of Porto, Porto, Portugal; ^3^Eating Disorders Unit, Psychiatry Department, Hospital of Santa Maria, Faculty of Medicine, University of Lisbon, Lisbon, Portugal

**Keywords:** anorexia nervosa, duration of illness, clinical impairment, severe-enduring criteria, staging

In the published article, there was an error in the Funding statement. The funding statement for Eva Conceição was incorrectly displayed as “IF/01219/2014”. The correct Funding statement appears below.

